# Correlation of Postoperative Outcomes According to the Amount of Prostatic Tissue Removed in Patients Undergoing Transurethral Resection of the Prostate

**DOI:** 10.7759/cureus.34451

**Published:** 2023-01-31

**Authors:** Ömer Turgut, Ahmet Erbagcı, Omer Bayrak, Ilker Seckiner, Sakıp Erturhan, Haluk Sen, Mehmet Ozturk

**Affiliations:** 1 Urology, Besni State Hospital, Adıyaman, TUR; 2 Urology, Gaziantep University Medical Faculty, Gaziantep, TUR

**Keywords:** lower urinary tract symptoms, transurethral resection of prostate, amount of tissue resected, prostate surgery, benign prostatic hyperplasia

## Abstract

Objective: The present study assesses the effect of the proportion of tissue resected during transurethral resections of the prostate (TUR-P) on lower urinary tract symptoms (LUTS) and other parameters in patients with a benign prostatic obstruction (BPO).

Materials and methods: Forty-three patients who underwent TUR-P between 2018 and 2021 were assessed prospectively. The patients were divided into two groups according to the percentage of tissue removed (group 1 <30%, group 2 >30% resection). Age, prostate volume, amount of resected tissue, operative time, length of hospital stay, duration of catheterization, International Prostate Symptom Score (IPSS), quality of life score (QoL), maximum urinary flow rate (Qmax), and serum prostate-specific antigen (PSA) (ng/dl) at preoperative and postoperative three months were recorded.

Results: The percentage of tissue removed was 22.2% vs. 48.4% (p = 0.001), IPSS reduction was 77.7% vs. 83.3% (p = 0.048), QoL improvement was 77.2% vs. 84.8% (p = 0.133), Qmax increase was 171.3% vs. 193.5% (p = 0.032), and serum PSA decrease was 56.4% vs. 69.2% (p = 0.049) in groups 1 and 2, respectively. In addition, the operative time was 38.5 vs. 53.6 min (p = 0.001), the length of hospital stay was 2.0 vs. 2.4 days (p = 0.001), and the duration of catheterization average was 4.1 vs. 4.9 days (p = 0.002).

Conclusion: Resections of at least 30% of prostatic tissue can provide a significant improvement in the symptoms and parameters related to benign prostatic obstruction, while resections of less than 30% of prostatic tissue can effectively reduce urinary symptoms and improve the quality of life in older adult patients with comorbidities who require shorter operating times.

## Introduction

Benign prostatic hyperplasia (BPH) is one of the most common health problems in aging men and is a histological diagnosis that is evident in almost every long-living man (90% of men by the age of 90 years) [1]. In a study examining the prevalence of lower urinary tract symptoms (LUTS), the pathology was found to begin and progress at 40-79 years of age [2]. According to this study, 13% of men aged 40-49 years and 28% of men over 70 years of age had moderate to severe difficulty urinating due to BPH.

Surgical treatment is often recommended for patients who do not benefit from medical treatment or who develop BPH-related complications (dysfunctional bladder, renal dysfunction, prostatic hematuria, and bladder stones) [3]. Transurethral resection of the prostate (TUR-P) has long been considered the standard for surgical treatment as it provides more than 90% improvement in voiding as well as significant improvement in quality of life in patients with the prostate volume of <80 g and BPH-associated LUTS in 10-year follow-up [4]. The standard TUR-P technique recommended by Nesbit involves the complete removal of the adenomatous tissue within the surgical capsule [5]. Studies in the literature reporting special cases have stated that partial resection (unilateral adenoma and median lobe if present) or removal of only the obstructing adenoma would be sufficient [6]. While the amount of tissue resected during TUR-P has decreased in recent years, there is still a lack of consensus on the amount of tissue that should be resected during TUR-P.

This prospective study assesses the amount of prostatic tissue resected and postoperative outcomes in patients undergoing TUR-P for BPH, and predicts the amount of tissue that should be resected to relieve LUTS related to BPH.

## Materials and methods

This study was conducted after gaining the approval of the Gaziantep University Faculty of Medicine (GUFM) Clinical Research and Ethics Committee (Decision date: 03.07.2019 and No. 2019/263).

Study participants

A total of 43 patients who underwent TUR-P for moderate-to-severe LUTS due to BPH between 2018 and 2021 were assessed prospectively.

Patients with moderate-to-severe LUTS due to BPH despite medical treatment, an International Prostate Symptom Score (IPSS) of >8, Qmax of <15 ml/sec, benign prostate biopsy, if performed, no serum prostate-specific antigen (PSA) and digital rectal examination (DRE) findings of malignancy, were included in the study. Patients who preoperatively had prostate or bladder cancer, urethral stricture, neurogenic bladder, bladder dysfunction, and those on medication that might lead to voiding dysfunction were excluded.

The study patients were assessed on the proportion of prostatic tissue removed, with those undergoing a <30% resection assigned to group 1 (n = 20) and >30% to group 2 (n = 23).

Surgical technique and sample collection

Preoperatively, all patients were evaluated based on their medical history, DRE, uroflowmetry, serum PSA test, complete urinalysis, complete blood count, biochemistry, and coagulation tests. The patients' symptoms, IPSS, and quality of life scores (QoL) were recorded [7]. Prostate volume was measured preoperatively by transabdominal ultrasonography (TAUS) using the formula: (anterior-posterior diameter) × (transverse diameter) × (sagittal diameter) × π/6.

All patients were operated on by the same surgeon. Regional cleaning and appropriate draping in the lithotomy position were performed under spinal or general anesthesia, and adenoma in the transitional zone and the median lobe, if any, were resected using an Olympus 26-Fr continuous-flow resectoscope and a standard electrode (Olympus Winter & Ibe GmbH, Hamburg, Germany) with isotonic saline irrigation. After bleeding control, a three-way catheter was inserted for continuous irrigation. Operative times were recorded. Prostatic tissue specimens were filtered thoroughly after the surgical procedure, weighed on a sensitive balance and recorded in grams (g). Since formaldehyde may cause a tissue weight loss of 10-13%, the specimens were placed in formaldehyde after weighing.

Follow-up

Complications that developed during surgery or at follow-up were noted and classified according to the modified Clavien-Dindo system [8]. The length of postoperative hospital stay and duration of urethral catheterization were recorded. All patients were called for evaluation at postoperative three months and Qmax values and serum PSA levels of the patients were measured. The patients were asked to complete the IPSS and QoL questionnaires.

The proportion of tissue removed was calculated using the formula: weight of tissue resected × 100/prostate volume on the preoperative ultrasonography (US).

Statistical analysis

SPSS for Windows (Version 11.0., SPSS Inc., Chicago, USA) was used for the statistical analysis, with data expressed as an arithmetic mean and standard deviation. A chi-square distribution test was used to calculate categorical variables and a Mann-Whitney U test to compare means. A confidence interval of 95% (p < 0.05) was considered statistically significant.

## Results

Preoperative assessment

The mean age of the 20 patients in group 1 was 69.5 ± 9.2 years, and that of the 23 patients in group 2 was 67.8 ± 10.2 years (p = 0.592). There was no statistically significant difference in the mean prostate volumes of group 1 and group 2 preoperatively, although it was lower in group 1 (60.9 ± 30.1 cm^3^ vs. 77.7 ± 35.08 cm^3^, p = 0.081). Preoperatively, group 2 had a higher IPSS score (27.2 ± 3.03 vs. 31.17 ± 2.69, p = 0.001), a lower Qmax (7.95 ± 2.76 ml/sec vs. 6.21 ± 2.79 ml/sec, p = 0.030), and a greater decrease in QoL (4.55 ± 0.6 vs. 5.34 ± 0.6, p = 0.001) than group 1. Preoperatively, the mean serum PSA was 5.71 ± 5.9 ng/ml in group 1 and 9.1 ± 9.5 ng/ml in group 2 (p = 0.059) (Table 1).

**Table 1:** Preoperative data. p < 0.05, n: number of patients, IPSS: International Prostate Symptom Score, QoL: quality of life, Qmax: maximum urinary flow rate, PSA: prostate-specific antigen.

	Group 1 (n = 20)	Group 2 (n = 23)	p-value
Age (years)	69.5 ± 9.2	67.8 ± 10.2	0.592
Prostate volume (cm^3^)	60.9 ± 30.1	77.7 ± 35.0	0.081
IPSS	27.2 ± 3.0	31.1 ± 2.6	0.001
QoL	4.5 ± 0.6	5.3 ± 0.6	0.030
Qmax (ml/sec)	7.9 ± 2.7	6.2 ± 2.7	0.001
PSA (ng/ml)	5.7 ± 5.9	9.1 ± 9.5	0.059

Postoperative assessment

The mean amount of prostatic tissue resected was 14.3 ± 9.6 g (22.2 ± 5.73%) in group 1 and 39.4 ± 25.7 g (48.4 ± 15.7%) in group 2 (p = 0.001, p = 0.001). There was no difference in the improvement in quality of life scores between the two groups (77.2% ± 17.1% vs. 84.8% ± 15.1%, p = 0.133). The percentage of IPSS reduction and the increase in Qmax were higher in group 2 (77.7 ± 8.4 vs. 83.3% ± 12.1, p = 0.048) (171.3 % ± 142.2 vs. 193.5 % ± 125.9, p = 0.032).

Postoperatively, the PSA level was 2.2 ± 2.4 ng/ml in group 1 and 2.6 ± 2.4 ng/ml in group 2, and the percentage PSA decrease was higher in group 2 (56.4 ± 20.1 and 69.2 ± 11.83%, respectively) (p = 0.049). Group 2 had statistically significantly longer operative time, longer hospital stay, longer urethral catheterization, and higher percentage of hemoglobin reduction (p = 0.001, p = 0.00, p = 0.002, p = 0.037, respectively) (Table 2).

**Table 2:** Postoperative data. p < 0.05, n: number of patients, IPSS: International Prostate Symptom Score, QoL: quality of life, Qmax: maximum urinary flow rate, PSA: prostate-specific antigen, Hgb: hemoglobin.

	Group 1 (n = 20)	Group 2 (n = 23)	p-value
Amount of tissue resected (g)	14.3 ± 9.6	39.4 ± 25.7	0.001
IPSS	6.0 ± 2.3	5.0 ± 3.5	0.206
QoL	1.0 ± 0.72	0.8 ± 0.8	0.436
Qmax (ml/sec)	17.5 ± 3.6	17.1 ± 6.1	0.287
PSA (ng/ml)	2.2 ± 2.4	2.6 ± 2.4	0.284
Hgb (mg/dl)	12.0 ± 2.3	12.2 ± 1.4	0.706
Operative time (min)	38.5 ± 7.4	53.6 ± 11.1	0.001
Length of hospital stay (days)	2.0 ± 2.4	2.4 ± 1.2	0.001
Duration of catheterization (days)	4.1 ± 2.6	4.9 ± 1.6	0.002

Postoperative blood transfusion was administered to two (4.6%) of the 43 study patients due to bleeding (Clavien II)-one in group 1 and the other in group 2. Uncomplicated urinary tract infection (UTI) occurred in four (20%) group 1 patients and four (17.3%) group 2 patients, totaling eight (18.6%) patients (Clavien I). Membranous urethral stricture developed in one (5%) group 1 patient and in the bulbar urethral stricture in one (4.3%) group 2 patient, and the stricture was relieved in these patients using a cold urethrotome knife under local anesthesia (Clavien IIIa). There was no statistically significant difference in the complications between the groups (p = 0.778).

## Discussion

The TUR-P surgical approach was first defined in the 1930s and remains the most popular and optimum approach to surgical treatment in patients with obstruction-related symptoms and a prostate volume of 30-80 g [9]. Although the standard TUR-P technique is described as the complete removal of the adenoma in the surgical capsule, there is a lack of guidelines on the percentage of tissue that should be removed for successful outcomes that will relieve the obstructive presentation.

The study by Liu et al. reported no correlation between the percentage of prostatic tissue removed from the transitional zone following TUR-P and the surgical outcome [10]. Among the studies supporting palliative or partial TUR-P, Aagaard et al. [11] reported that obstruction relief alone (minimal TUR-P) may be sufficient without resection down to the capsule, while Agrawal et al. demonstrated that the short-term outcomes of the removal of the unilateral transitional zone and, if present, the median lobe were comparable to standard TUR-P [6].

Among the studies involving the calculation of the amount of prostate removed, Green et al. [12] reported a mean amount of 25.6 g in a case series of 432 patients, compared to 14.1 g reported by Yoon et al. [13] and 36.6 g by Seçkiner et al. [14]. A review of the literature also reveals studies reporting tissue removal rates of 28-78% [15-18], indicating clearly the difference in the amounts of tissue removed by different surgeons. In the present study, the standard TUR-P technique was performed on 43 patients in a single center by the same surgeon, and a mean of 27.7 g and 36.2% of prostatic tissue was removed from all patients.

In the present study, group 1 comprised patients who had less than 30% of tissue resected (n = 20), and group 2 consisted of patients who had more than 30% of tissue resected (n = 23). In our review of the literature, Antunes et al. [18] were considered to be the only previous study with similarities to the present study, although the authors divided their patients into three groups according to the percentage of resected tissue, being group 1 <30% (n = 23), group 2 30-50% (n = 43), and group 3 >50% (n = 22). Considering IPSS, the study by Antunes et al. reported a mean value decrease of 16.7 in group 1, 16.6 in group 2, and 18.4 in group 3 (p < 0.001), with a greater increase noted in group 3 (p = 0.561). In the current study, the mean decrease in IPSS value was 21.2 (77.7%, p = 0.001) in group 1, and 26.1 (83.3%, p = 0.001) in group 2. Although there was a significant decrease in IPSS in both groups, the decrease was statistically more significant in group 2 (p = 0.048). These data suggest that the percentage of IPSS improvement increases as the rate of resected tissue increases. Antunes et al. [18] reported a decrease of value 3.1 in group 1, 3.9 in group 2, and 4.2 in group 3, with the most significant decrease being in group 3 (p=0.046) about the QoL score. In the present study, the preoperative and postoperative scores were 4.5 vs. 1.0 (77.2%, p = 0.001) in group 1, and 5.3 vs. 0.8 (84.8%, p = 0.001) in group 2, respectively. The improvement in QoL was significant in both groups, and there was no statistically significant difference between them (p = 0.133).

Serum PSA is normally used to screen for prostate cancer. The study by Bohnen et al., involving a large patient series, established a strong relationship between PSA and prostate volume [19]. This study reported a prostate volume of >30 ml in patients with a PSA value of >1.5 ng/ml, with a positive predictive value of 78%. Antunes et al. [18] found no statistically significant variation in PSA in the <30% resection group, while there was significant variation in the PSA in the >50% resection group. In the current study, the preoperative and postoperative PSA values were 5.7 vs. 2.2 (56.4%) in group 1 and 9.1 vs. 2.6 (69.2%) in group 2, respectively. Although there was a significant change in both groups, the decrease in PSA was statistically more significant in group 2 than in group 1 (p = 0.049). This finding, similar to other studies in the literature, suggests that the amount of prostate tissue removed and the decrease in PSA are correlated and that the PSA variation could be used to predict the success of surgery in patients undergoing TUR-P.

Yoon et al. reported a 116% increase in Qmax in a sample of 49 patients with a resection rate of 28% [13]. In another study, a 197% increase in Qmax was recorded with a resection rate of approximately 38% [17]. In the present series, the preoperative and postoperative values were 7.9 vs. 17.5 ml/sec (171.3%, p = 0.001) in group 1 and 6.2 vs. 17.1 ml/sec (193.5%, p = 0.001) in group 2, respectively. Although there was a significant improvement in Qmax in both groups, the rate of change in group 2 was higher than that in group 1, and the rate of improvement in Qmax increased as the resection rate increased (p = 0.032).

Surgery for BPH is often performed on middle-to-advanced-aged patients, which sometimes makes it difficult to deal with complications and is associated with significant mortality and morbidity. The study by Mebust et al. found an increased risk of complications in patients with a prostate of >45 g and an operative time of >90 min [20]. Common early complications include mortality, retention after catheter removal, bleeding requiring transfusion, urinary infection, clot retention, and TUR syndrome, while common late complications are secondary hemorrhage, bladder neck, and urethral stricture. In our series, two (4.6%) of 43 patients were given a postoperative blood transfusion due to bleeding-one in group 1 and the other in group 2. Uncomplicated urinary tract infections developed in four (20%) group 1 patients and four (17.3%) group 2 patients, totaling eight (18.6%) patients. Urethral stricture developed in one (5%) group 1 patient and in one (4.3%) group 2 patient. The complication rate in our study patients is similar to that reported in an earlier meta-analysis [21]. There was no statistically significant difference in the complication rates of our study groups.

Operative time is important for the development of complications. Wilhelm et al. resected 51% of tissue in a mean of 55.3 min in patients with a prostate volume of <70 cc and 54% of tissue in a mean of 82.6 min in those with a prostate volume of >70 cc [22]. Bach et al., on the other hand, resected 11 g of tissue in 37 min in their group with a prostate volume of <40 cc, 26 g of tissue in 57 min in the group with 40-79 cc, and 40 g of tissue in 61.5 min in the group with >80 cc [23]. In the present study, the resection time was 38.5 min in group 1 and 53.6 min in group 2 (p = 0.001). Similar to previous studies, it would seem that greater proportions or amounts of tissue removed lead to longer operations, as in group 2. The mean length of hospital stay was 2.1 days (2.0 days in group 1 and 2.4 days in group 2, p = 0.001), and the mean duration of catheterization was 4.5 days (4.1 days in group 1 and 4.9 days in group 2, p = 0.002). Based on these findings, it can be concluded that the length of hospital stay and the duration of catheterization increase in proportion to the rate of tissue resected.

Study limitations

In the present study, we used TAUS to calculate the prostate volume of patients. Studies have shown transrectal ultrasonography (TRUS) and MRI to be more accurate than TAUS in revealing the anatomy and measuring the size of the prostate [24]. Although it can be considered a limitation of our study, we preferred TAUS as it is easily accessible, non-invasive, easily applicable, and inexpensive. Our study presents only short-term (three months) outcomes, while studies reporting five-year outcomes after TUR-P involving large patient series have reported a re-TUR-P requirement in 5% of patients [25,26]. Considering this information, the number of patients requiring reoperation due to the development of secondary hemorrhage, urethral stricture, and residual adenoma after three months could not be assessed, which can be considered a further limitation of our study.

The standard TUR-P technique recommends the resection of all existing adenomas, and this method has been widely adopted by surgeons. Candidates for TUR-P are often older adults and are likely to have comorbidities, leading to increased morbidity and mortality rates due to prolonged operation times and a greater amount of tissue removed [27,28]. The findings of both the present and previous studies suggest that a large amount of tissue removal through TUR-P provides a more pronounced improvement in symptom score and voiding curve, although we believe that the resection of the obstructing adenoma alone and the removal of a small amount of tissue can lead to a reduction in complications associated with surgery as well as an adequate improvement in quality of life and symptom scores.

## Conclusions

According to our findings, a resection rate of at least 30% provides a significant improvement in symptoms and the parameters related to benign prostatic obstruction. Resections of at least 30% of prostatic tissue can adequately relieve lower urinary tract symptoms and improve the quality of life in older adult patients with comorbidities who require shorter operating times. Owing to the limited number of similar studies in the literature, there is a need for studies of similar design that present long-term outcomes.
